# Effects of Cu Pollution on the Expansion of an Amphibious Clonal Herb in Aquatic-Terrestrial Ecotones

**DOI:** 10.1371/journal.pone.0164361

**Published:** 2016-10-13

**Authors:** Liang Xu, Zhen-Feng Zhou

**Affiliations:** College of Resource and Environment, Qingdao Agricultural University, Qingdao, China; Shandong University, CHINA

## Abstract

Physiological integration can enhance the performance of clonal plants in aquatic and terrestrial heterogeneous habitats and associated ecotones. Similar to nutrients, pollutants may be transported among connected ramets via physiological integration. Few studies have examined the expansion of amphibious clonal plants from terrestrial to aquatic environments, particularly when the local water supply is polluted with heavy metals. A greenhouse experiment was conducted using the amphibious plant *Alternanthera philoxeroides* to determine whether Cu can spread among clonal plants and examine the corresponding effects of this pollution on the expansion of clonal plants in aquatic-terrestrial ecotones. Ramets from the same clonal fragments were rooted in unpolluted soil and polluted water at five different levels. The responses of the ramets in terrestrial and aquatic habitats were quantified via traits associated with growth, morphology and Cu accumulation. The results indicated that ramets in soil and water significantly differed in nearly all of these traits. The expansion of populations from terrestrial to polluted aquatic habitats was facilitated by stem elongation rather than new ramet production. The accumulated Cu in polluted ramets can be horizontally transported to other ramets in soil via connected stolons. In terms of clonal growth patterns, variations in Cu pollution intensity were negatively correlated with variations in the morphological and growth traits of ramets in polluted aquatic habitats and unpolluted soil. We concluded that Cu ions are distributed among the clones and accumulated in different ramet tissues in heterogeneous habitats. Therefore, we suggest that Cu pollution of aquatic-terrestrial ecotones, especially at high levels, can affect the growth and expansion of the whole clones because Cu ions are shared between integrated ramets.

## Introduction

Many exotic invasive plants are clonal species, and clonal growth characteristics, such as physiological integration are associated with the ability of plants to adapt to foreign environments, compete with local plants and colonize associated habitats [1–4]. In ramets, the development of horizontal runners, such as rhizomes, roots and stolons, facilitates the translocation of water, nutrients, carbohydrates, etc. [5–8]. Thus, physiological integration is an important ability that facilitates the survival and growth of new ramets in stressful heterogeneous environments and improves the performance of the entire clone [9–11]. Certain clonal invasive plants are also amphiphytes [12–14]. During the entire life cycle, clonal growth assists these plants in resisting heterogeneous stress and expanding populations between terrestrial and aquatic habitats [14–17].

However, physiological integration does not always benefit entire clones under environmental stress [17–19], and plants suffering from long-term or severe stress might lose fitness and terminate support to dependent ramets [18]. The distribution of disease [17] and transmission of toxic pollutants, such as heavy metals, via connected runners might impose extra costs on the entire clonal system [9, 11, 20, 21]. The balance between benefits and costs depends on the extent of integration in different species and the environmental stress [20].

Aquatic ecosystems are important receptors of municipal sewage and industrial wastewater; thus, large areas of these ecosystems have been endangered because of heavy metal pollution. Considering the long-term and non-degradable characteristics of heavy metals, heavy metal pollution in natural water and wetlands caused by human activities has become a worldwide environmental problem [22–24]. Upon entering the environment, in organic metal ions display persistent toxicity that directly inhibits vegetation growth and threatens animal and human health throughout the food chain. A number of studies have focused on the effects of various environmental factors, such as temperature, pH and organic pollutants, on heavy metal toxicity to plants, and several models (e.g., BLM, FIAM, GSIM and related models) have been developed to estimate the bioactivity of heavy metals to aquatic and terrestrial organisms [9, 25, 26]. These studies and models have primarily focused on individual plants as objects to assess the bioaccumulation and biotoxicity of heavy metals; however, only a few studies have focused on the toxic responses of amphibious clonal plants at the population level and the expansion of these plants in polluted habitats [9, 27]. In aquatic-terrestrial ecotones, heterogeneous pollution caused by different types or concentrations of heavy metals presents a challenge to clonal plants because of the potential costs to donor ramets and the transport of pollutants. Therefore, heavy metal pollution in natural waters might hamper the rapid expansion of amphibious invasive plants from terrestrial to aquatic habits and the subsequent invasion of these plants into aquatic environments.

Phenotypic plasticity and adaptation to local circumstances are two alternative strategies of plants to accommodate heterogeneous environments, and they are particularly apparent in invasive plants [28]. Nevertheless, the population genetic variation of certain invasive species outside of their native environments is extremely low [29–32]. Thus, natural selection might have less of an effect, and local adaptation is less likely to occur. Moreover, phenotypic plasticity should be the preferred strategy for invaders in new environments [30].

Phenotypic plasticity in plants is generally related to the morphological (particularly leaf and stem traits), physiological (e.g., physiological integration, division of labor) and ecological plasticity of individual plants [2, 33–35]. Clonal plants interconnected with runners represent strongly integrated systems in which the morphological and physiological traits of individual ramets are highly dependent on the connected ramets [15]. There are clear trade-off responses among different phenotypic structures of plants growing in patchy environments that reflect the optimization of biomass investments in growth and reproduction as well as population survival and functional maintenance [2, 33]. In polluted environments, toxic substances (e.g., heavy metals) might increase the costs and reduce the fitness of physiologically integrated clones by altering the water, nutrient and energy flows and promoting the translocation of toxic pollutants [9, 20, 21]. Thus, heavy metal pollution in natural waters should be associated with the invasiveness of amphibious clonal plants between terrestrial and aquatic habitats. The significance of this association likely depends on the type, concentration and speciation of heavy metals in the natural habitats.

In the present study, we showed how heavy metal pollution in water affects the invasive capacity and associated phenotypic plasticity of the amphibious invasive plants in aquatic-terrestrial ecotones. We specifically addressed the following questions: (1) How do the ramets of plants expand in heterogeneous habitats, and do the growth, morphological and Cu accumulation traits vary among habitats? (2) To what extent do pollutants affect the phenotypic plasticity of plants and the functional correlations between adaptation and expansion? (3) How does the plasticity of growth, morphological and Cu accumulation traits vary in response to pollution stress? To achieve our objectives, the seedlings of *Alternanthera philoxeroides*, a common amphibious invasive species with clonal growth traits, were used to simulate the invasion of plants from terrestrial to aquatic environments at different pollution intensities.

## Material and Methods

### Ethics statement

*Alternanthera philoxeroides* is a common amphibious clonal plant in China. We collected fragments of *Alternanthera philoxeroide* from an uncultivated area along the bank of the Moshui River in Qingdao, Shandong Province, China. Thus, specific permission was not required for the collection of this herbaceous plant or to visit the location where we collected the samples. In addition, we collected only fragments from these sites. We confirm that the fieldwork did not involve any endangered or protected species.

### Focal species and study site

*Alternanthera philoxeroide* (Mart.) Griseb (Amaranthaceae) is an amphibious perennial herb originally from South America [36]. This herb has invaded more than thirty countries located in tropical and/or temperate areas worldwide [12, 29]. The stems produce new ramets, opposite leaves and adventitious or perennial roots at any node. The reproduction of this plant rarely involves the generation of viable seeds and primarily depends on vegetative propagation via stems or perennial roots. All of the ramets integrated with stolon runners form a complete clone system, in which resource sharing plays an important role in the plant's adaptation and invasion via physiological integration [12, 14, 19, 29, 37]. As an alien invader in China, *A*. *philoxeroides* is widely distributed in terrestrial (e.g., crop lands, roadside) and aquatic (e.g., still water, river flow) habitats and junctions. In the wild, this weed expands from terrestrial to aquatic environments and *vice versa* [16, 17]. Compared with the strong invasiveness and wide distribution of this plant, the genetic variation of *A*. *philoxeroides* across China is minimal according to molecular data [12, 29, 31, 32], suggesting that phenotypic plasticity is crucial for the life cycle and invasive expansion of this plant species.

### Experiment and measurements

On 21 June 2014, clonal fragments of *A*. *philoxeroides* were collected along the banks of the Moshui River in Qingdao, Shandong Province, China. The background value of Cu in this area was 16.43±2.59 mg kg^-1^(means ± SD), which was much lower than the Class I (35 mg kg^-1^) of Environmental quality standard for soils (GB15618-1995). The plants were transplanted and propagated in a greenhouse located at Qingdao Agricultural University, China. In the greenhouse, the mean diurnal temperature during the experiment was 25°C and the average humidity was 40%. All the plants were collected from a relatively small area. Because of the low genetic variation of the species across China [12, 29, 31, 32], the significance of genetic differentiation on the results can be ignored.

**Fig 1:**
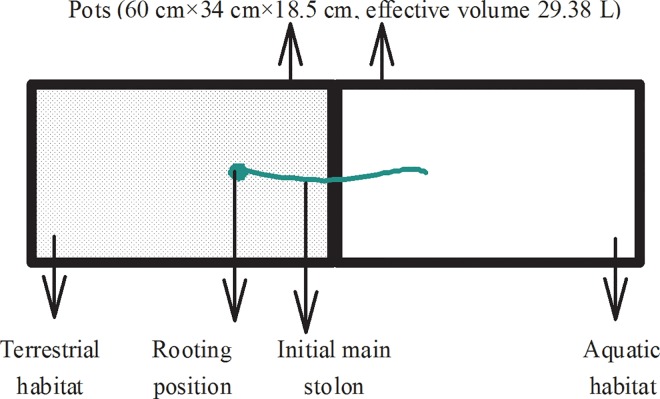
Schematic illustration describing the experimental design. Two pots of the same size were used to simulate the terrestrial and aquatic habitats. The terrestrial habitat was simulated using a 1:1 mixture of soil and sand and four grams of solid slow-release fertilizer. The aquatic habitat was simulated using tap water and different levels of Cu in sulfate form. The small clonal fragments consisted of a perennial root and a single stolon with an apex and were rooted from the edge at a distance of one fourth the pot length. The design is described in the experiment and measurements section of the Materials and Methods.

On 25 May 2015, small similarly sized fragments that included a perennial root, and a single stolon with an apex were separated from their clonal populations and rooted in pots (60 cm×34 cm×18.5 cm, effective volume 29.38 L). Each pot was filled with a 1:1 mixture of soil and sand and four grams of solid slow-release fertilizer (16N-11P_2_O_5_-11K_2_O-3MgO + trace elements, 3–4 months, Osmocote Exact, Scotts International B.V, Heerlen, the Netherlands) to simulate a terrestrial environment. The rooting position from the edge was one fourth of the pot length (Fig 1). After 14 days, the average total length of the stem reached 18.52±0.27 cm, and the initial main stolon had grown past the pot edge. Totally 35 plants were selected and randomly assigned for further processing. Another pot simulating an aquatic environment was filled with tap water (Fig 1). Cu was added as a sulfate solution (CuSO_4_·5H_2_O) into the tap water (the background value of Cu was 0.60 ± 0.11 μmol L^-1^) at different doses to simulate a polluted water environment. Five Cu concentration gradients (0, 0.5, 1, 2 and 3 mmol L^-1^) were initially used. This design of concentration gradients ensured that there were enough living ramets in the end of the experiment [27]. During the experiment, the plants in the soil were regularly watered. The water surface elevations in the pots simulating aquatic habitats were maintained at nearly the same height.

Every ten days, the total length of the stems and total number of new ramets generated from the nodes were recorded, totally involving six growth stages (viz. 60 days) and seven censuses. Plants in the terrestrial and aquatic habitats were harvested on August 8, 2015. The fifth and sixth internodes of the stolons in the soil (counting from the initial rooting position) and water (counting from the edge of the pots) were severed from the clone. In this experiment, it was observed that the primary, secondary and tertiary ramets came out successively from the perennial roots or the initial main stolons. The selected stolons all belonged to the primary branching ramets. The fifth and sixth internodes were in the middle of the stolons, which were mature enough for the measurements. The external diameter, inner diameter, leaf length and leaf width of the fifth and sixth internodes were measured. In addition, the mean length of the perennial roots in soil and adventitious roots in water were measured and calculated. Subsequently, the plants were separated into leaves, roots and stems using sharp scissors. The roots were carefully washed. The dry biomass was determined after the leaves, roots and stems were dried in a stove at 70°C for 48 h.

After measuring the dry biomass, the Cu contents of the different organs were analyzed. Samples of the leaves, roots and stems were ground using a ball mill (DECO-PBM-V-4L, Changsha, Hunan Province, China) and dried to a constant weight. The homogenized samples were placed in Teflon crucibles (effective volume 100 ml; Changji, Dongtai, Jiangsu Province, China) containing 25 ml of H_2_O_2_/HNO_3_ at a ratio of 1:4 (*v*/*v*) for eight hours. The crucibles were sealed with steel cans and placed in a stove for one hour at 80°C. After hydrolysis, the crucibles were heated on an electrical heating panel (MWJ-3020, Wuxi, Jiangsu Province, China) at 120°C for two hours to remove excess acid from the solution. The Cu contents in the extracts were analyzed via inductively coupled plasma-optical emission spectrometry (Optima 8000, Perkin Elmer, Massachusetts, USA).

### Data processing

The mean absolute growth rates of the total stem length (G_L_) and total number of new ramets (G_NR_) during the six stages were calculated using the data obtained from seven measurements. For example, for stage 1, the measurement from census 1 to 2 was calculated as (X_2_-X_1_) / 10, where X is the trait value measured at each census. The leaf shape index (LSI), which reflects the phenotypic variation of the leaves, was calculated as the ratio of the leaf length to width. The stem area ratio (SAR) was calculated as the square of the ratio of the inner diameter to the external diameter.

The plasticity of the plants in response to terrestrial and aquatic habitats was calculated as follows:

$$Plasticity=({VALUE}_{aquatic}-{VALUE}_{terrestrial})/{VALUE}_{terrestrial}$$ Eq 1

where VALUE _aquatic_ and VALUE _terrestrial_ refer to trait measurements (e.g., length, number of new ramets or Cu accumulation traits) of different constituents of plants living in aquatic and terrestrial habitats, respectively. Using this method, independent measurements of plasticity in the same plant systems were paired prior to calculating the plasticity to facilitate the statistical comparison of the plasticity of plants under different Cu stress conditions. In this equation, plasticity is considered relative rather than absolute, and the percentage variation in the different traits of plants in aquatic habitat relative to those in the terrestrial habitats is calculated [33]. Positive results for plasticity show larger trait values for plants in aquatic habitats than for those in terrestrial environments. Stronger plasticity values, regardless of positive or negative direction, indicate a higher degree of variation in plasticity.

### Data analysis

A two-way ANOVA was used to examine the effects of habitat and Cu pollution on the growth, morphological and Cu accumulation traits. A one-way ANOVA was used to examine the differences in plasticity under different Cu pollution conditions. Prior to the analysis, the data were assessed for equality of variance using Levene’s test and for normality using the Shapiro–Wilk test. The regression equations in the figures were generated using Sigmaplot 12.5 software (Systat Software Inc., Erkrath, Germany). SPSS 21 (IBM Inc., Armonk, New York, USA) was used for all of the statistical analyses. *P*< 0.05 was used as the significance level. The data showed in the figures were presented as the means ± SE without transformation.

## Results

**Fig 2:**
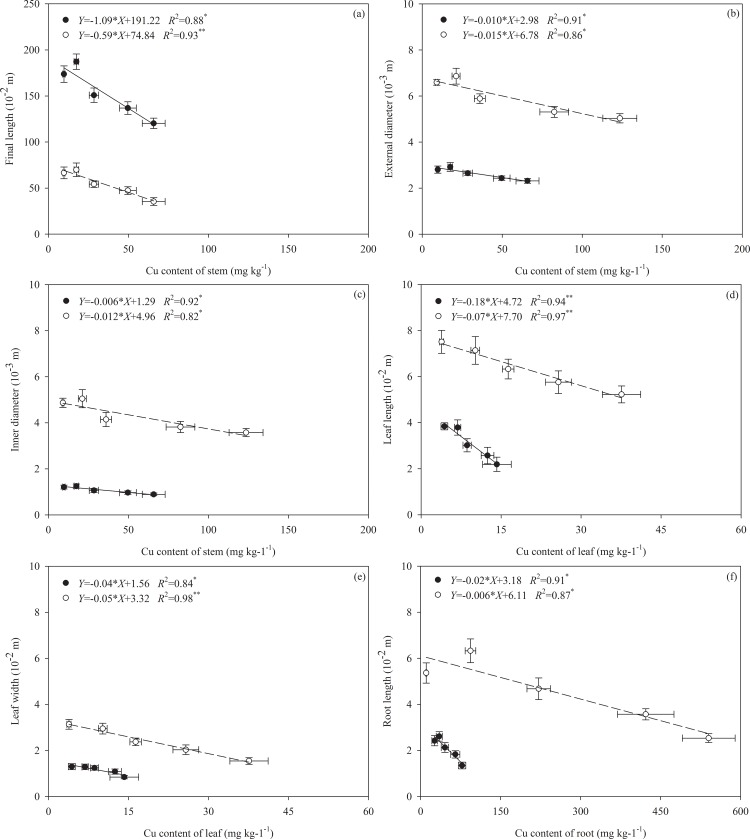
Relationships among the Cu content of the stem and the final length (a), external diameter (b), and inner diameter (c); Cu content of the leaf and the leaf length (d) and leaf width (e); and Cu content of the root and the root length (f) of *Alternanthera philoxeroides* in terrestrial habitats (black circles; unbroken lines) and aquatic habitats (white circles; broken lines). Regression equations, coefficient of determination (R^2^) and significance level (*, *P* < 0.05, **, *P* < 0.01) are shown. Values are indicated as the means ± SD.

**Fig 3:**
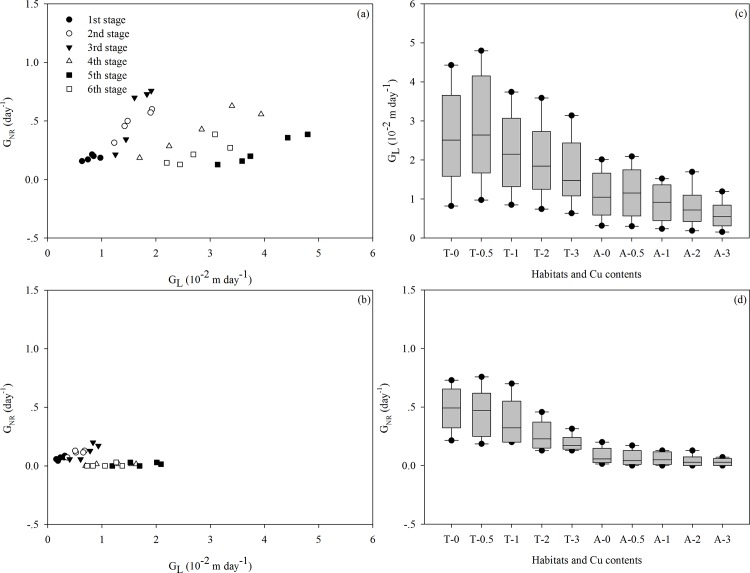
Growth rate of the total length (G_L_) and growth rate of new ramets (G_NR_) of *Alternanthera philoxeroides* in terrestrial habitats (a) and aquatic habitats (b) across six stages. The box-plot graphs show the variation in G_L_ (c) and G_NB_ (d) of the plants in terrestrial habitats (T) and aquatic habitats (A) under different Cu pollution, and the data for the stages are grouped together.

**Fig 4:**
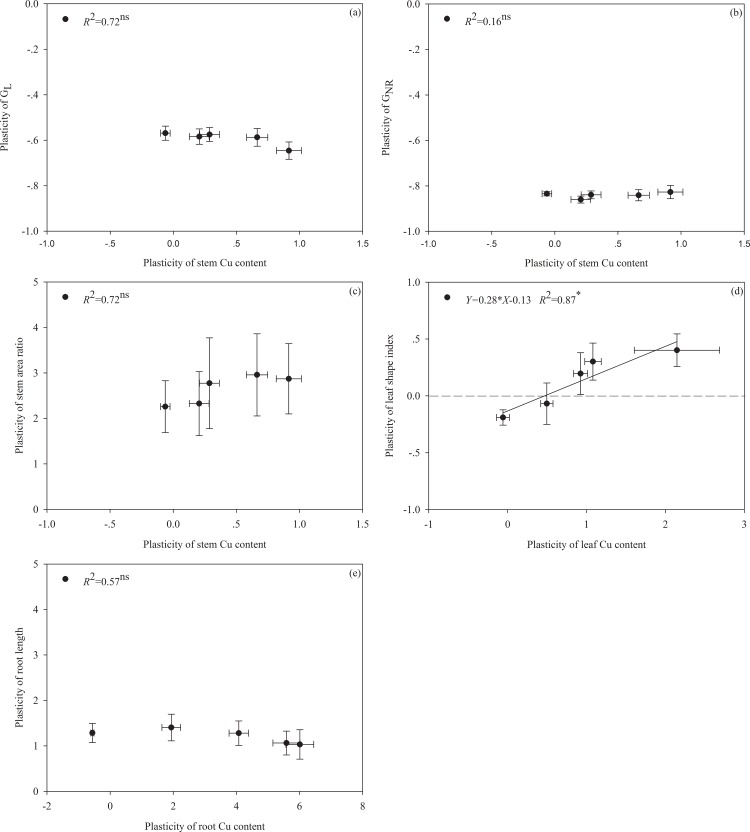
Relationship between the plasticity of the mean stem Cu content of the plants throughout the entire experiment and the plasticity of the mean growth rate of the total length (G_L_) (a), mean growth rate of new ramets (G_NR_) (b), stem area ratio (c), leaf shape index (d) and root length (e);between the plasticity of the mean leaf Cu content and the plasticity of the leaf shape index; and between the plasticity of the mean root Cu content and the plasticity of root length. Regression equations (unbroken line), coefficient of determination (R^2^) and significance level (*, *P*< 0.05; ^ns^, *P*> 0.05) are indicated. The broken line represents the expected relationship when there is no plasticity in the trait depicted on the y axis.

**Fig 5:**
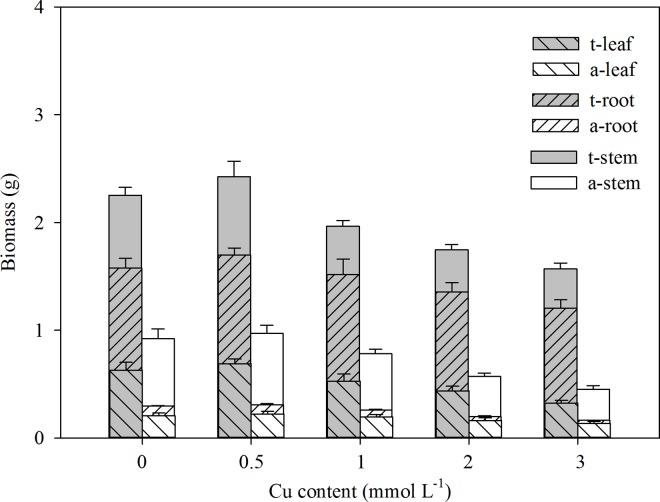
Leaf (right slash), root (left slash) and stem (blank) biomass of the plants in the terrestrial (gray bars) and aquatic habitats (white bars) under different Cu pollution conditions. Data are presented as the means ± SE without transformation.

Although belonging to the same clone, the ramets from the terrestrial and aquatic habitats performed differently in nearly all of the measured traits (Table 1; Figs 2–5).

**Table 1:** Results of the two-way ANOVA showing the effects of Cu pollution (*P*; *df* = 4) and different habitats (*H*; *df* = 1) on the growth, morphological and Cu accumulation traits of *Alternanthera philoxeroides*.

Growth							Cu accumulation				
	Total mass	Leaf mass fraction	Stem mass fraction	Root mass fraction				Leaf	Root	Stem	Total
P	**10.04**	0.20	1.43	0.36			P	**70.23**	**115.53**	**165.69**	**165.69**
H	**246.08**	0.02	**718.84**	**1842.27**			H	**57.92**	**173.93**	**22.36**	**22.36**
P×H	0.14	**7.34**	0.55	**13.06**			P×H	**6.98**	**37.13**	**3.97**	**3.97**
Morphology											
	Length	Leaf length	Leaf width	LSI	E-diameter	I-diameter	SAR	Root length	G_L_	G_L1_	G_L2_
P	**19.60**	**8.68**	**13.86**	0.33	**13.76**	**8.59**	0.40	**23.84**	**19.19**	**14.32**	**15.55**
H	**568.94**	**169.87**	**163.82**	0.18	**869.34**	**995.49**	**145.08**	**182.19**	**348.15**	**691.81**	**450.26**
P×H	2.01	0.17	**2.65**	1.59	0.94	0.53	0.07	1.52	2.01	1.50	**2.80**
	G_L3_	G_L4_	G_L5_	G_L6_	G_NR_	G_NR1_	G_NR2_	G_NR3_	G_NR4_	G_NR5_	G_NR6_
P	**8.54**	**16.38**	**6.24**	**4.01**	**51.78**	1.12	**6.57**	**17.43**	1.28	**4.18**	**4.18**
H	**149.99**	**126.71**	**149.39**	**101.58**	**1340.63**	**58.15**	**244.08**	**163.64**	**175.60**	**1546.10**	**1546.10**
P×H	0.26	2.44	0.68	0.02	**10.62**	0.13	0.83	**4.85**	0.41	1.96	1.96

Morphology abbreviations are described in the experiment and measurements section of the Materials and Methods. *F* values are indicated, and the bold values indicate *P*< 0.05. Data were natural logarithm transformed prior to the analyses.

Ramets rooted in soil had larger growth rates for both stem elongation (G_L_) and new ramet production (G_NR_) than those rooted in water for each of the six growth periods (Table 1; Fig 3A and 3B), which resulted in longer total stem lengths and consistently broader occupation in terrestrial habitats (Table 1; Figs 2A and 3). Except for the total stem length, the other morphological traits of the ramets in the soil, including the external and inner stem diameter, leaf length and width, and root length, were smaller than those of the ramets in the water (Table 1; Fig 2B–2F). The SAR, which was calculated based on the external and inner diameter and correlated with floatability, was much larger in the aquatic environment (Table 1; Fig 4C). Similar to the stem length, the total biomass of the ramets was smaller in the aqueous conditions than the soil conditions (Table 1; Fig 5). Moreover, the aquatic environment induced higher biomass allocation to the stems at the expense of the roots, whereas the terrestrial environment showed an opposite performance trend (Table 1; Fig 5).

The mean Cu concentrations in the leaves, roots and stems of all of the ramets increased with increasing Cu levels in the water habitat, which suggested that Cu was transported among the clones (Table 1; Fig 2). The highest Cu concentration was detected in the roots located in polluted water (Table 1; Fig 2F). Only a fraction of the Cu (ranging from *c*.5% to *c*.11%) was accumulated in the leaves (Fig 2D). Because of the different biomass investments observed in the terrestrial and aquatic habitats, an average of*c*.67% Cu was deposited in the stems of the ramets in water during transport and an average of*c*.70% Cu was stored in the perennial roots in the soil (Fig 2D and 2F).

The plants under different concentrations of Cu pollution showed significant differences with respect to many traits except for the stem mass fraction, LSI and SAR (Table 1; Figs 2–4). A significant interactive effect between the different habitats and Cu pollution was observed in the leaf and root mass fraction, leaf width, Cu accumulation and G_NR_ (Table 1). In particular, the effect of pollution on the LSI showed opposite trends between the different habitats. Although exposure to the pollutant decreased the leaf length and width, the leaf width appeared to be more sensitive to Cu stress. Cu contamination induced a maximum decrease in leaf length and leaf width of *c*. 43% and *c*. 35% in the terrestrial habitats and *c*.30% and *c*.51% in the aquatic environments, respectively (Fig 2D and 2E). These results supported the hypothesis that the habitat-induced plasticity (Eq 1) differed significantly between aqueous and terrestrial habitats (Table 2). Interestingly, under varying pollution intensities, only the plasticity of the LSI was positively correlated with the plasticity resulting from Cu accumulation in the leaves (Fig 4).

**Table 2:** Results of the one-way ANOVA showing the differences in plasticity (as calculated with Eq 1) among the terrestrial and aquatic habitats (*df* = 1) for the morphological, growth and mechanical traits of *Alternanthera philoxeroides*.

	Biomass	Length	ED	ID	SAR	Root length	Leaf length	Leaf width	LSI	G_L_	G_NR_	Root Cu	Stem Cu	Leaf Cu
Pollution	2.65	2.11	0.38	0.09	0.16	0.34	1.11	0.94	**2.78**	0.79	0.37	**66.26**	**24.50**	**19.01**

Abbreviations are described in the experiment and measurements section of the Materials and Methods. Length is the final total length of all stems. Root Cu, stem Cu and leaf Cu indicated the Cu contents in these tissues. *F* values are indicated, and the bold values indicate *P*< 0.05. Data were natural logarithm transformed prior to the analyses.

In general, the effects of Cu pollution on stem elongation and new ramet production changed over time. Shoot elongation increased by *c*. 59% on average in the first two stages, but this difference decreased to *c*. 37%in the next three stages and to *c*. 19% in the last stage (Table 1; Fig 3A and 3C). The growth rate of new ramets, which represents a strategy among invasive plants with clonal traits for occupying new habitats, decreased from *c*. 89% to *c*. 6% throughout all stages (Table 1; Fig 3B and 3D). In terms of the clonal growth patterns, variations in the intensity of Cu pollution were negatively correlated with variations in the most morphological and growth traits of the ramets in the polluted aquatic environments and had negative effects on the connected ramets in the terrestrial habitats (Table 1; Figs 2–5). A lot of ramets in the polluted aquatic environments died as a result of the excessive accumulation of Cu during the experiment. In the end, all the clones rooting in the soil and parts of the ramets in the polluted water were alive in each treatment. Particularly, the Cu pollution did not significantly decrease the total numbers of new ramets in the water (13 of the control *vs*. 11 of the highest Cu level; *P* = 0.996). In the terrestrial environment, the light Cu pollution (0.5 mmol L^-1^) did not affect the production of new ramets (*P* = 0.999). However, with the increasing Cu levels, the numbers of new ramets reduced from 58 (0 mmol L^-1^) to 52 (1 mmol L^-1^; *P*< 0.01), 47 (2 mmol L^-1^; *P*< 0.001) and 36 (3mmol L^-1^; *P*< 0.001). Under direct Cu stress, less biomass was allocated to the roots in the polluted water, which might alleviate the absorption of toxic substances (Table 1; Fig 5). Biomass allocation to the leaves increased by *c*. 23% on average between the highest Cu levels and the control, but biomass allocation to the stem kept close to *c*. 68%(Table 1; Fig 5). On the other hand, in unpolluted soil, additional biomass was invested in the perennial roots from *c*. 42% to *c*. 56% with increasing Cu levels (Table 1; Fig 5). Increasing Cu pollution did not affect the biomass investment in leaves but decreased the investment in the stems by *c*. 20% (Table 1; Fig 5)

## Discussion

### Phenotypic plasticity among heterogeneous habitats

Phenotypic plasticity and genetic differentiation are the two major strategies of alien invasive plants for adapting to heterogeneous habitats. As a result of the low genetic variations of *A*. *philoxeroides* across China [12, 29, 31, 32], phenotypic plasticity should be the primary strategy among these plants when invading and colonizing in heterogeneous environments. Environmental variation is reflected in the plasticity of the plants as exhibited in morphological, physiological, and mechanical traits [33]. Considering the large span from terrestrial to aquatic environments, the tissue structure of amphibious plants should simultaneously possess terrestrial and aquatic traits to accommodate soil and water habitats [14, 16, 17]. We observed that the phenotypic plasticity of *A*. *philoxeroides* influenced its adaptability to different habitats during expansion. Generally, *A*. *philoxeroides* extend populations to heterogeneous habitats via the elongation of stolons and production of new ramets (viz. branching). The former strategy is common, whereas the latter is selective. Furthermore, phenotypic directional trends were primarily determined via environmental factors affecting the plants, particularly aqueous environmental factors.

In the present study, the growth rates of the total stem length (G_L_) and number of new ramets (G_NR_) in water were slower than those in soil. Previous studies have shown that the branching intensity usually increases with increasing resources [38, 39]. Thus, shorter internodes and larger branching intensities increase the efficiency of resource acquisition [28, 40].Resource distribution and levels in terrestrial habitats are generally more heterogeneous relative to those of aquatic environments. For *A*. *philoxeroides*, the rapid elongation rate of the stem and increased production of new ramets in soil might benefit the plants by increasing their ability to forage for favorable patches and avoid polluted areas. In aquatic habitats, population dispersal is more likely to rely on the elongation of stems and not on the sprouting of new ramets, which might be associated with the maintenance of floatability. The elongation of new stolons floating in water directly increases the water surface contact, which is associated with variations in buoyancy. Moreover, lower investments in new ramets, particularly erect shoots, likely reduces the potential risk of submergence resulting from excessive biomass.

The stem area ratio, root length, leaf length, width and correlated leaf shape index are all morphological attributes of *A*. *philoxeroides* that displayed strong compatibility and were used as indicators of adaptability. The larger inner cavity of hollow stems increased the floatability in water and was beneficial for stem ventilation and vegetative growth maintenance. In soil, bulky perennial roots are responsible for resource fixation, resource storage, winter survival and new adventitious bud production (personal observation); however, these roots easily draw water and inorganic minerals from the aquatic environment. Thus, only the adventitious root system is developed in aqueous environments so that resources are more easily concentrated. In addition, the adventitious root system can also draw some oxygen, partially increase floatability, support fragments, and buffer stems against the mechanical impact of currents [41]. For the variations in leaves, the smaller leaf length, width and associated leaf shape index of plants grown in soil rather than in water indicates an adaptive response to water conservation. The leaves of plants grown in water may be subjected to the flooding stress. As a result of the slow gas diffusion in water, the flooding can directly lead to oxygen depletion, especially in the submerged plants. When suffering from flooding, the plants may respond to submergence by increasing leaf length and/or width, which may shift the leaves above the water surface [34, 42].

### Cu translocation and risk sharing among clones

Irrespective of the level of Cu pollution in water, the presence of interconnected stolons is likely to guarantee the transport of resources from ramets rooting in soil to support the growth and dispersal of ramets growing in aquatic habitats [17]. Physiological integration has the potential to contribute to the expansion of amphibious clonal species in aquatic-terrestrial ecotones [14]. Under Cu pollution, *A*. *philoxeroides* might also suffer from distribution of toxicity via stolon connections. The types of habitats and the stabilization of heavy metal pollution affect the activity of free toxic metals [9, 43]. In the present study, the activity and corresponding toxicity of Cu ions was positively correlated with the Cu concentrations in the water.

For most plant species, heavy metals, including Cu, primarily assimilate and accumulate in the roots and subsequently restrict the upward translocation to other organs via vascular tissues [13]. In the clonal plant *A*. *philoxeroides*, Cu was distributed in the roots, stems and leaves at different levels. The extremely low Cu concentrations in the soil compared with that of the polluted water likely did not cause high Cu accumulation in the ramets rooting in soil, suggesting that the Cu was distributed among the clones in different living patches. In the polluted water, Cu moved from the adventitious roots and was horizontally redistributed to the leaves and stems. In addition, a portion of the Cu was exported via the horizontal stolons to the ramets in unpolluted soil. Consequently, Cu pollution was basipetally distributed to the belowground roots and horizontally and acropetally distributed to the leaves and stems.

Toxic effects might appear when the amount of Cu accumulated in the tissues exceeds certain levels depending on the tolerance of the species and activity of the Cu ions [43, 44]. In the ramets of *A*. *philoxeroides*, only a fraction of Cu (less than 10%) was reserved in the leaves, even if the plant was rooted in soil or polluted water. On average, *c*. 67% Cu was accumulated in the stems of the ramets grown in polluted water, whereas up to *c*. 70% Cu was exported and deposited in the perennial roots of the ramets grown in soil. The stem elongation, new ramet production, leaf size, root length and other traits of the ramets in both the terrestrial and aquatic environments were severely inhibited at Cu concentrations above 1 mmol L^-1^. Certain clonal grass species grown under patchy pollution conditions have been shown to randomly distribute offspring ramets in both polluted and unpolluted patches without active selection and avoidance, and this process generates a similar population density in different patches [9]. In small water areas, it is hard for elongated stolons to escape from heavy metal stress when the plants are relatively homogeneously distributed; thus, the plants cannot relocate to more favorable conditions. In this case, the dispersal of pollutant stress among all of the clones through physiological integration can redistribute the risk and alleviate the toxicity experienced by ramets in direct contact with the pollutant. Considering the benefit-cost balance, shorter thinner stems and reduced production of new ramets would not benefit the physiological integration of nutrients, suggesting that the growth of individual ramets of *A*. *philoxeroides* was relatively independent.

Consistent with an intraclonal division of labor, which is one of the basic characteristics of clonal plants, polluted environments might result in less biomass allocation to roots, which would result in proportionally less absorption of the pollutant, whereas ramets in unpolluted soil might invest more biomass in the roots to draw adequate nutrients to share among all of the clones [9, 35]. Less biomass allocation to the roots, shorter root lengths, lower elongation rates of stems and reduced production rates of new ramets in polluted water might mitigate the risks of Cu stress to ramets in both water and soil environments. However, the higher distribution of biomass to the roots of ramets grown in soil implies that *A*. *philoxeroides* employs compensatory investments to the roots to draw nutrients and store pollutants. Cu stress on the leaves reduced the leaf length and width, but the LSI varied in the water and soil conditions, indicating that the leaf width was more sensitive to Cu pollution.

## Conclusions

As a typical amphibious clonal plant, phenotypic plasticity helps the ramets of *A*. *philoxeroides* distribute between terrestrial and aquatic habitats. Physiological integration is beneficial to ramets that receive nutrients at the cost of the ramets that contribute nutrients [14, 17, 45]. Under heavy metal stress, Cu could spread among the clones living in aquatic-terrestrial ecotones via horizontal stolons. Risk sharing might alleviate the accumulated toxicity to the ramets in direct contact with the pollutant, but it will inflict stress to the physiologically integrated ramets in unpolluted patches. Most of the exported Cu was accumulated in the stolons and perennial roots of the ramets in the soil. Consequently, the ramet growth, morphology and Cu accumulation traits in all habitats were under Cu stress and showed similar trends. At a global scale, the clonal plant species with larger benefits of physiological integration are not more invasive, but these in which recipient clonal parts living in unfavorable environments benefit more from physiological integration are more invasive [46].With the continuous development of the social economy, the heavy metal concentrations in the environments have been accelerating annually. Except for the resource translocation in the clones, the risk sharing between integrated ramets can also benefit the invasive species *A*. *philoxeroides* in the polluted habitats to maintain the global invasion success. However, it is likely that the invasiveness of the population is negatively correlated with the intensity of Cu pollution in water.

There are some limitations in the current study. Although our finding presents an interesting perspective on simultaneous risk spreading and resource sharing via physiological integration, we stress that more work is needed to investigate the potential cooperation and antagonism between heavy metal elements and nutrients in amphibious clonal plants. Besides, the conclusions were mainly deduced from the morphological and growth changes. Thus, the other traits, for example, the mechanical and anatomical traits, should be involved to better illustrate the underlying mechanisms.

## Supporting Information

S1 Table The average values of the morphological, growth and Cu accumulation traits of *Alternanthera philoxeroides*. (DOCX)
