# Supplementary material for: Effects of Cu Pollution on the Expansion of an Amphibious Clonal Herb in Aquatic-Terrestrial Ecotones
Source: PLoS One. 2016 Oct 13;11(10):e0164361. doi: 10.1371/journal.pone.0164361 (PMC5063404; doi:10.1371/journal.pone.0164361)
Supplement: S1 Table — (DOCX) [file pone.0164361.s001.docx]

**S1 Table. The average values of the morphological, growth and Cu accumulation traits of *Alternanthera philoxeroides*.**

|  | Growth |  |  |  | Cu accumulation | |  |  |  |  |
| --- | --- | --- | --- | --- | --- | --- | --- | --- | --- | --- |
|  | Total mass (g) | Leaf mass fraction | Root mass fraction | Stem mass fraction | Leaf (mg kg^-1^) | Root (mg kg^-1^) | Stem (mg kg^-1^) | | Total (mg kg^-1^) | |
| T-0 | 2.25±0.21 | 0.28±0.01 | 0.42±0.02 | 0.30±0.02 | 4.36±0.65 | 26.16±5.07 | 9.71±0.73 | | 33.13±4.41 | |
| T-0.5 | 2.42±0.22 | 0.29±0.02 | 0.42 ±0.03 | 0.28±0.04 | 6.85±0.59 | 34.79±5.76 | 17.46±1.45 | | 50.84±4.79 | |
| T-1 | 1.96±0.23 | 0.27±0.01 | 0.49±0.02 | 0.24±0.03 | 8.62±0.77 | 45.52±6.95 | 28.48±2.98 | | 65.34±14.67 | |
| T-2 | 1.74±0.13 | 0.25±0.01 | 0.52±0.03 | 0.23±0.03 | 12.46±1.20 | 65.08±8.58 | 49.65±5.22 | | 80.35±6.43 | |
| T-3 | 1.56±0.13 | 0.21±0.01 | 0.56±0.03 | 0.24±0.03 | 14.19±2.69 | 77.69±7.08 | 65.79±7.17 | | 93.64±4.22 | |
| A-0 | 0.92±0.11 | 0.23±0.02 | 0.09±0.01 | 0.68±0.02 | 3.83±0.38 | 10.43±1.22 | 9.06±0.67 | | 7.40±1.07 | |
| A-0.5 | 0.97±0.10 | 0.23±0.02 | 0.08±0.01 | 0.69±0.02 | 10.17±0.83 | 93.58±9.97 | 21.16±2.53 | | 22.20±1.64 | |
| A-1 | 0.78±0.06 | 0.25±0.02 | 0.07±0.01 | 0.68±0.02 | 16.33±1.09 | 221.53±22.14 | 36.01±3.44 | | 33.07±2.42 | |
| A-2 | 0.57±0.06 | 0.28±0.02 | 0.05±0.01 | 0.67±0.02 | 25.74±2.43 | 422.17±52.96 | 82.54±8.98 | | 47.07±7.14 | |
| A-3 | 0.45±0.05 | 0.30±0.02 | 0.04±0.01 | 0.66±0.02 | 37.58±3.58 | 540.21±49.48 | 123.52±10.60 | | 50.75±7.03 | |

|  | Morphology | | | |  |  |  |  |  |  |  |  |  |  |  |  |  |  |
| --- | --- | --- | --- | --- | --- | --- | --- | --- | --- | --- | --- | --- | --- | --- | --- | --- | --- | --- |
|  | Total stem length (cm) | Leaf length (cm) | Leaf width (cm) | LSI | E-diameter (mm) | | I-diameter (mm) | | SAR | | Root length (cm) | | G_L_ (cm day^-1^) | | G_L1_ (cm day^-1^) | | G_L2_ (cm day^-1^) | |
| T-0 | 173.73±8.98 | 3.84±0.15 | 1.30±0.13 | 3.14±0.35 | 2.81±0.16 | | 1.21±0.09 | | 0.19±0.02 | | 2.42±0.22 | | 2.94±0.17 | | 0.82±0.05 | | 1.93±0.08 | |
| T-0.5 | 187.23±8.31 | 3.79±0.33 | 1.29±0.12 | 3.12±0.41 | 2.92±0.19 | | 1.25±0.09 | | 0.20±0.03 | | 2.61±0.20 | | 3.18±0.16 | | 0.97±0.05 | | 1.90±0.10 | |
| T-1 | 150.84±7.82 | 3.02±0.28 | 1.24±0.09 | 2.59±0.40 | 2.65±0.10 | | 1.06±0.09 | | 0.16±0.02 | | 2.13±0.21 | | 2.47±0.15 | | 0.85±0.05 | | 1.48±0.09 | |
| T-2 | 136.89±6.86 | 2.57±0.36 | 1.08±0.12 | 2.71±0.63 | 2.44±0.10 | | 0.97±0.08 | | 0.16±0.02 | | 1.83±0.16 | | 2.23±0.14 | | 0.74±0.05 | | 1.42±0.10 | |
| T-3 | 120.27±5.61 | 2.19±0.31 | 0.85±0.08 | 2.63±0.29 | 2.32±0.10 | | 0.89±0.06 | | 0.15±0.02 | | 1.35±0.15 | | 1.90±0.10 | | 0.64±0.04 | | 1.23±0.10 | |
| A-0 | 66.55±6.35 | 7.51±0.50 | 3.13±0.21 | 2.43±0.15 | 6.59±0.12 | | 4.87±0.20 | | 0.55±0.05 | | 5.36±0.44 | | 1.27±0.12 | | 0.32±0.03 | | 0.68±0.07 | |
| A-0.5 | 69.90±7.37 | 7.14±0.60 | 2.95±0.23 | 2.59±0.40 | 6.86±0.35 | | 5.04±0.39 | | 0.57±0.09 | | 6.33±0.51 | | 1.34±0.15 | | 0.30±0.02 | | 0.66±0.06 | |
| A-1 | 54.27±3.59 | 6.33±0.43 | 2.38±0.16 | 2.69±0.16 | 5.89±0.21 | | 4.14±0.31 | | 0.50±0.06 | | 4.68±0.47 | | 1.04±0.07 | | 0.24±0.03 | | 0.52±0.06 | |
| A-2 | 47.30±4.16 | 5.76±0.49 | 2.03±0.21 | 3.03±0.44 | 5.31±0.23 | | 3.82±0.24 | | 0.56±0.10 | | 3.57±0.24 | | 0.91±0.09 | | 0.19±0.01 | | 0.51±0.06 | |
| A-3 | 35.40±4.17 | 5.23±0.37 | 1.55±0.14 | 3.58 ±0.41 | 5.03±0.20 | | 3.57±0.18 | | 0.53±0.07 | | 2.53±0.19 | | 0.68±0.08 | | 0.16±0.02 | | 0.36±0.04 | |

|  | Morphology |  |  | |  |  |  | |  |  |  | |  |  |  | |  |  | |  | |  |  | |  | |  |  | |  | |  |  | |  | |  |  | |  | |  |  | |  | |  |  |  |
| --- | --- | --- | --- | --- | --- | --- | --- | --- | --- | --- | --- | --- | --- | --- | --- | --- | --- | --- | --- | --- | --- | --- | --- | --- | --- | --- | --- | --- | --- | --- | --- | --- | --- | --- | --- | --- | --- | --- | --- | --- | --- | --- | --- | --- | --- | --- | --- | --- | --- |
|  | G_L3_ (cm day^-1^) | | | G_L4_ (cm day^-1^) | | | | G_L5_ (cm day^-1^) | | | | G_L6_ (cm day^-1^) | | | | G_NR_ (day^-1^) | | |  | | G_NR1_ (day^-1^) | | |  | | G_NR2_ (day^-1^) | | |  | | G_NR3_ (day^-1^) | | |  | | G_NR4_ (day^-1^) | | |  | | G_NR5_ (day^-1^) | | |  | | G_NR6_ (day^-1^) | | |  |
| T-0 | 1.83±0.16 | | | 3.40±0.24 | | | | 4.43±0.29 | | | | 3.09±0.33 | | | | 0.58±0.04 | | | | | 0.21±0.03 | | | | | 0.60±0.04 | | | | | 0.73±0.08 | | | | | 0.63±0.09 | | | | | 0.36±0.06 | | | | | 0.39±0.16 | | | |
| T-0.5 | 1.91±0.09 | | | 3.94±0.25 | | | | 4.80±0.40 | | | | 3.37±0.35 | | | | 0.54±0.02 | | | | | 0.19±0.03 | | | | | 0.57±0.03 | | | | | 0.76±0.07 | | | | | 0.56±0.07 | | | | | 0.39±0.07 | | | | | 0.27±0.04 | | | |
| T-1 | 1.60±0.09 | | | 2.84±0.20 | | | | 3.74±0.33 | | | | 2.69±0.38 | | | | 0.45±0.02 | | | | | 0.20±0.03 | | | | | 0.50±0.07 | | | | | 0.70±0.10 | | | | | 0.43±0.07 | | | | | 0.20±0.06 | | | | | 0.21±0.01 | | | |
| T-2 | 1.45±0.12 | | | 2.24±0.26 | | | | 3.59±0.25 | | | | 2.44±0.46 | | | | 0.31±0.01 | | | | | 0.17±0.02 | | | | | 0.46±0.02 | | | | | 0.34±0.06 | | | | | 0.29±0.09 | | | | | 0.16±0.04 | | | | | 0.13±0.05 | | | |
| T-3 | 1.25±0.12 | | | 1.70±0.19 | | | | 3.14±0.16 | | | | 2.20±0.30 | | | | 0.22±0.01 | | | | | 0.16±0.02 | | | | | 0.31±0.06 | | | | | 0.21±0.05 | | | | | 0.19±0.05 | | | | | 0.13±0.02 | | | | | 0.14±0.03 | | | |
| A-0 | 0.84±0.08 | | | 1.55±0.23 | | | | 2.01±0.28 | | | | 1.26±0.17 | | | | 0.10±0.01 | | | | | 0.09±0.03 | | | | | 0.13±0.02 | | | | | 0.20±0.03 | | | | | 0.01±0.01 | | | | | 0.03±0.02 | | | | | 0.03±0.02 | | | |
| A-0.5 | 0.94±0.19 | | | 1.63±0.24 | | | | 2.09±0.38 | | | | 1.38±0.26 | | | | 0.08±0.01 | | | | | 0.07±0.03 | | | | | 0.11±0.01 | | | | | 0.17±0.02 | | | | | 0.01±0.01 | | | | | 0.01±0.01 | | | | | 0.00±0.00 | | | |
| A-1 | 0.78±0.10 | | | 1.31±0.31 | | | | 1.52±0.14 | | | | 1.06±0.20 | | | | 0.07±0.01 | | | | | 0.07±0.02 | | | | | 0.11±0.03 | | | | | 0.13±0.04 | | | | | 0.01±0.01 | | | | | 0.03±0.02 | | | | | 0.00±0.00 | | | |
| A-2 | 0.61±0.08 | | | 0.90±0.13 | | | | 1.69±0.32 | | | | 0.83±0.13 | | | | 0.05±0.01 | | | | | 0.04±0.02 | | | | | 0.13±0.02 | | | | | 0.06±0.03 | | | | | 0.01±0.01 | | | | | 0.00±0.00 | | | | | 0.00±0.00 | | | |
| A-3 | 0.41±0.06 | | | 0.70±0.16 | | | | 1.19±0.23 | | | | 0.73±0.10 | | | | 0.04±0.01 | | | | | 0.06±0.02 | | | | | 0.07±0.02 | | | | | 0.06±0.02 | | | | | 0.00±0.00 | | | | | 0.00±0.00 | | | | | 0.00±0.00 | | | |

The ‘T’ represents the terrestrial habitat while the ‘A’ represents the aquatic habitat. The 0, 0.5, 1, 2 and 3 represent the different levels of Cu.
